# Generative AI and unstructured audio data for precision public health

**DOI:** 10.1038/s44401-025-00022-7

**Published:** 2025-06-02

**Authors:** James Anibal, Adam Landa, Hang Nguyen, Veronica Daoud, Tram Le, Hannah Huth, Miranda Song, Alec Peltekian, Ashley Shin, Lindsey Hazen, Anna Christou, Jocelyne Rivera, Robert Morhard, Jacqueline Brenner, Ulas Bagci, Ming Li, Yael Bensoussan, David Clifton, Bradford Wood

**Affiliations:** 1 Center for Interventional Oncology, Radiology and Imaging Sciences, NIH Clinical Center, Bethesda, USA; 2 Computational Health Informatics Lab, Oxford Institute of Biomedical Engineering, University of Oxford, Oxford, UK; 3 Oxford University Clinical Research Unit, Ho Chi Minh City, Vietnam; 4 Morsani College of Medicine, University of South Florida, Tampa, FL USA; 5 College of Engineering, University of South Florida, Tampa, FL USA; 6 Department of Computer Science, McCormick School of Engineering, Northwestern University, Evanston, IL USA; 7 National Library of Medicine, National Institutes of Health, Bethesda, MD USA; 8 Feinberg School of Medicine, Northwestern University, Chicago, IL USA

**Keywords:** Data processing, Machine learning, Predictive medicine

## Abstract

In this study, transcribed videos about personal experiences with COVID-19 were used for variant classification. The o1 LLM was used to summarize the transcripts, excluding references to dates, vaccinations, testing methods, and other variables that were correlated with specific variants but unrelated to changes in the disease. This step was necessary to effectively simulate model deployment in the early days of a pandemic when subtle changes in symptomatology may be the only viable biomarkers of disease mutations. The embedded summaries were used for training a neural network to predict the variant status of the speaker as “Omicron” or “Pre-Omicron”, resulting in an AUROC score of 0.823. This was compared to a neural network model trained on binary symptom data, which obtained a lower AUROC score of 0.769. Results of the study illustrated the future value of LLMs and audio data in the design of pandemic management tools for health systems.

## Introduction

Audio data for health (“audiomics”) has been recognized as a promising tool for advancing digital medicine, potentially enabling low-cost, non-invasive methods for clinical tasks including rapid diagnostics and patient monitoring^1^. However, much work must be done to improve the multimodality of audio datasets, which may include voice, speech, and language biomarkers (Table 1). The majority of past studies, particularly in infectious disease medicine and pandemic management, have primarily focused on the use of standardized acoustic data to identify voice changes, losing the potential insights contained within freely spoken language.

**Table 1:** Description of key information sources within audio data

Aspect	Definition	Key Components	Examples
Voice	The sound produced by vibration of the vocal folds	Pitch, Loudness, Quality, Resonance	Hoarseness, Breathiness, Nasality
Speech	The physical production of sounds to form words	Articulation, Phonology, Fluency	Pronunciation of sounds, sound patterns in words, stuttering
Language	A system of communication, often using words	Phonology, Morphology, Syntax, Semantics, Pragmatics	Vocabulary use, grammar, sentence structure, expressing meaning

Unsurprisingly, there were many attempts to build voice/sound AI models for diagnostic tasks involving COVID-19. In one example, COVID-19 breath sounds were detected via unique time and frequency domain patterns^2^. AI technologies trained on cough sounds have also been deployed on a smartphone app for COVID-19 detection, and a binary classifier was able to differentiate COVID-19 speech from normal speech based on scripted data from telephone calls^3–8^. In another example, the spectral features of speech in asymptomatic patients with and without COVID-19 yielded a true positive rate of 70%, though the models were trained on a small dataset^9^. A CNN model trained on forced-cough recordings from a small number of patients was able to recognize COVID-19 with high sensitivity, even in asymptomatic subjects^10^. There have also been initiatives to create COVID-19 voice repositories through crowdsourcing and online data mining. “Coswara” is a database of coughs, breathing, and voices reading standardized scripts (recorded/uploaded by volunteers)^11^. The samples were divided into COVID-19 (self-reported positivity) and control cohorts^11^. Researchers have used Coswara to train AI models for COVID-19 detection and variant classification, often obtaining high accuracies on binary datasets that typically excluded other respiratory illnesses^3,6,12–15^. Conversely, deep learning models trained on the “Sounds of COVID” crowdsourced dataset showed that the voice modality alone led to poor performance on COVID-19 screening tasks (0.61 AUC score)^16^. The COVYT dataset contains COVID-related videos from social media, with corresponding control samples from the same speakers^17^. However, past COVYT studies do not account for the significant differences in content between the positive and negative cohorts^17^.

While still relatively uncommon, AI models trained on unstructured audio data—such as free speech—have also shown promise in a variety of health applications, including voice-based clinical assessments^18^. Unlike standardized datasets, unstructured audio data allows for more natural interactions as patients discuss their health in their own words. One notable area of research focuses on the automated generation of electronic health records using voice recognition (i.e., ambient listening)^19^. Multiple AI scribes have been developed to transcribe and organize conversations between doctors and patients^19–21^. The resulting EHRs are then approved by clinicians, potentially reducing workload while improving the accuracy and quality of medical records. The application of AI to unstructured audio data (“free speech”) has also included diagnostic tasks, including for neurodegenerative diseases like Parkinson’s and Alzheimer’s, as well as speech impairments such as aphasia and dysarthria^22^.

Despite potential, many of these prior attempts have failed due to reliance on small, binary datasets, producing overfit models which do not generalize^8,16^. Because of such challenges, no quantifiable benefits were derived from AI models that were trained on voice/audio data collected during the COVID-19 pandemic. Simple screening models trained on reported symptoms were found to be equally effective^23^.

In this report, a pipeline was developed for tasks involving unscripted, real-world audio data and was compared with algorithms trained on basic symptom data in tabular form. This study applied large language models (LLMs) and weakly supervised deep learning methods to perform COVID-19 variant classification based on summarized audio transcripts extracted from online videos^24^. Variant classification is a key component of pandemic management: differences in viral strains were shown to have implications related to infectiousness and severity^25^. In comparison to other diagnostic methods that rely on lab results or images, AI tools trained on unstructured audio data may be more cost-effective for health systems—while also containing more nuanced insights than models trained on binary symptom data. Contributions were as follows:

1. **Development of a practical pipeline that could be implemented within the early phases of a pandemic**. Experiments were designed to simulate scenarios that may arise at the onset of future health crises. LLM-driven summarization was used for rapidly curating datasets, enabling the rapid training of variant classification models on a CPU. Results of this study demonstrated the value of hybrid digital health systems involving the application programming interfaces (API) of existing generalist AI tools and customized small models that can be deployed on local devices or with minimal cloud resources.
2. **Classification of viral variants through unstructured audio data**. In this novel application of neural network models, predictions were made based on speaker-reported symptoms and other health-related information from audio transcripts, without reliance on references to the date, vaccination status, past infections, or other factors that may have been indicators of variant status but were not causally related to any changes in the COVID-19 virus. After training on unstructured audio data, the neural network outperformed a similar model trained on binary representations of symptoms. The performance of this system (within a simulated emerging pandemic) matched the results of large studies involving digital survey data, further showing the potential value of unstructured audiomic data. Future systems may be built around free-speech audio recordings to augment data from limited EHR or other conventional mechanisms for information collection - while also providing additional insights found within correlated acoustic features.

In contrast to conventional voice AI, which is often solely reliant on acoustic features like fundamental frequency or jitter, the system presented in this report was designed to leverage language information contained in unscripted audio data recorded by speakers who were describing their experience with COVID-19. Such a system could be trained on crowdsourced data and perform inference on vast amounts of audio data from future pandemic situations, effectively supporting health systems.

## Results

In this study, transcribed descriptions of COVID-19 cases were summarized by the o1 LLM, removing noise and references to variables that may have co-occurred with specific prevalent variants but were unrelated to changes in disease pathology. This helped ensure an accurate simulation of an early-stage crisis in which only symptom information is available to train AI models for emergency deployment. The LLM-generated summaries were then visualized as word clouds in order to identify broad symptomatologic trends within the dataset that may be valuable in understanding the results of AI model predictions. Table 2 contains a specific example of the summarized audio transcripts used to obtain these insights.

**Table 2:** Example of a transcribed audio summary used for COVID-19 variant visualization and classification

The speaker experienced a range of covid-19 symptoms over a period of several days. Initially, they felt a scratchy throat and had a lingering cough, which worsened over time. they developed body aches, particularly severe in the legs, and experienced difficulty regulating their body temperature, with episodes of intense sweating and chills. the speaker also reported a fever, although they did not have a working thermometer to confirm it. as the days progressed, they experienced extreme fatigue and nausea, which persisted for several days. despite these symptoms, the speaker did not experience any breathing difficulties or require hospitalization. They noted that their symptoms were intense but short-lived, with each day bringing new challenges. By the end of the symptomatic period, they felt congested but had regained some energy and no longer had a fever.	

**Fig. 1:**
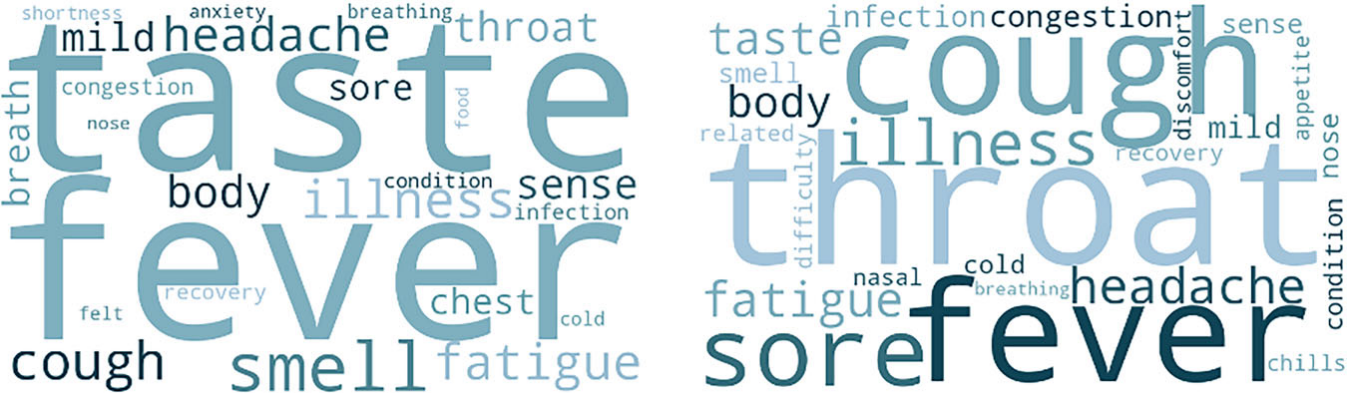
Results of transcript summary visualization. The word cloud on the left side displays features from LLM-generated summaries of audio transcripts in the pre-Omicron cohort. The word cloud on the right side displays features from LLM-generated summaries of audio transcripts in the Omicron cohort.

In Fig. 1 (left), results show that LLM summaries within the Pre-Omicron cohort often included references to symptoms such as the loss of taste and/or smell, which aligns with large-scale studies^26^. Many of the recurring keywords from Omicron summaries were upper respiratory symptoms like cough and sore throat – this transition also matched the findings in existing scientific literature (Fig. 1 – right)^26^.

The results shown in Fig. 1 emphasize the value of free-speech audio data in capturing clinically relevant information. Despite the use of low-cost, unstructured data from online sources, experimental outcomes aligned with large studies involving the prospective collection of standardized data^26^.

Following visualization, neural networks were trained to perform variant classification using embedded representations of health information in the transcripts, excluding data that could coincidentally indicate the variant (e.g., the date or testing methods). Nested k-fold cross-validation was used to evaluate the performance of the models. Across multiple experimental iterations, the audio AI model obtained a mean AUROC score of 0.823, indicating a moderately robust signal. The specificity of the model was 0.70 when the sensitivity value was calibrated to 0.80 (Table 3). Results were obtained using a neural network model trained on single CPU. The average time for training the neural network was ~3.7 s per split (the inference time was negligible). The second neural network, trained on vectors of binarized data (i.e., the presence or absence of symptoms), resulted in a lower AUROC score of 0.769 with a specificity of 0.60.

**Table 3:** Comparison of neural network models trained on summarized audio data and binary vectors indicating the presence or absence of symptoms

Data Type	AUROC	Specificity (@0.80 Sens.)	Sensitivity
Summarized Audio Transcripts	0.823	0.70	0.83
Binary Symptom Vectors	0.769	0.60	0.82

These results show that the embedded summaries of unstructured audio transcripts led to a superior performance on the variant classification task. This was in comparison with data from the same transcripts that was preprocessed more conventionally (Table 6) to align with simple “symptom-checking” systems. As seen in Table 3, there was a 5.4% difference in the AUROC score and a 10% difference in specificity at 0.80 sensitivity. DeLong’s test for comparison of correlated ROC curves resulted in a *p*-value of 0.0045, well below the accepted threshold for statistical significance (0.05)^27^.

## Discussion

This report shows that “free speech” audio data has potential value in pandemic settings or other public health applications when paired with powerful LLMs and neural networks. In this study, LLMs were used to generate summaries of lengthy audio transcripts from public online sources. Visualizations of these summaries elucidated similar insights to immense and costly research efforts. This detailed information was then used to train a neural network for variant classification (AUROC = 0.823), which outperformed a “symptom-checking” model trained on binary data extracted from the same transcripts but without the additional context of free speech. Moreover, these results were obtained in a simulation environment designed to replicate the early stages of a pandemic, showing the potential value of unstructured audio data for time-sensitive tasks like outbreak monitoring. This work may be impactful in compute-constrained healthcare systems where extensive EHR mining is infeasible, but patient-reported audio recordings may be collected easily, preprocessed by LLMs, and used to train task-specific models.

With further development, this may be applied in multiple different ways. Examples include the parsing of large audio repositories to identify samples related to a certain disease, the integration of audio data with EHR through LLM-driven preprocessing, and epidemiological surveillance tasks. For example, the pipeline could be used to process, anonymize, and analyze data from diverse online sources, resulting in much larger training datasets than those used in the study. This work also underscored the inherent multimodality of audio data, moving beyond simple acoustic biomarkers from voice and instead focusing on spoken language. Prospective studies, such as the hearai.org effort, could be run to enhance the multimodality of crowdsourced audio data, collecting spoken descriptions of health and acoustic variables from tasks like reading the Rainbow Passage^18,28^. This may enhance the value of audio-based AI models.

One additional future application of this audio-based AI pipeline may be within emergency departments (EDs). EDs are often high-volume settings with insufficient resources and time to meet the needs of every patient. Rapid assessment is critical for patient safety and public health. By facilitating the analysis of multimodal audio data, this pipeline may be used to predict key clinical insights—like shifts in infectious disease patterns. Developing rapid, non-invasive tools that can triage patients more efficiently could significantly enhance quality of care and trust in healthcare systems. For example, AI models trained on audio data could identify subtle patterns of symptoms that are unique to an emerging infectious disease, helping clinicians activate isolation protocols and forecast disease progression. In a diverse ED population, some patients may require more immediate and intensive care, while others can be safely observed or discharged. Integrating audio analysis within ED workflows may optimize resource utilization in high-stress environments, improving patient outcomes. However, these possibilities have been obstructed by the lack of new technologies that can adapt to the challenging environment—the work presented in this study may be a promising solution.

The impact of this work is currently speculative due to possible biases in the dataset and weak annotations. Variant status could not be confirmed serologically but was inferred based on the date of recording. The dataset was also developed based on the availability of online videos meeting the inclusion criteria (Table 4). These videos contained unverified claims about symptoms and disease severity—unintentional misreporting of these aspects may reduce model performance on downstream data collected from a clinical setting. Future work should involve the testing of “crowdsourced” AI models on data that was prospectively collected with corresponding gold-standard annotations. Additionally, due to the uncontrolled nature of public data, biases are likely present in this preliminary study. For example, audio recordings extracted from YouTube videos may skew towards a younger demographic, causing reduced performance amongst older patients who may exhibit different symptoms. There was also a lack of linguistic diversity—most of the speakers used English in the videos, leading to uncertainty about model performance in other languages and possibly limiting global health applications.

**Table 4:** Inclusion criteria for the cohorts of unstructured audio data defined in this study

Cohort	Criteria for Inclusion	#Videos	Example Title
Omicron variant	The speaker explicitly confirmed that they tested positive for COVID-19 on or after December 1^st^, 2021, and discussed their COVID-19 case.	303	I have Covid
Pre-Omicron variants	The speaker explicitly confirmed that they tested positive for COVID-19 on or before November 30^th^, 2021, and discussed their COVID-19 case.	404	A day in my life with COVID

Furthermore, implementation obstacles may arise in clinical settings. For example, the EDs are often overcrowded with patients, resulting in a loud, chaotic environment that may reduce the likelihood of collecting clear, uninterrupted audio data. Future work should involve the benchmarking and fine-tuning of speech processing models on noisy recordings containing detailed medical terminology. These types of domain-specific datasets paired with robust learning objectives may improve performance in environments like the ED.

Ultimately, digital health is understudied in the context of generative AI, deep learning, and multimodal audio data. However, the wide availability of such data and the advanced capabilities of models like o1 raise important new research questions. As audio/video applications rapidly increase in popularity, unscripted audio may be more available and inherently more diverse than conventional data, leading to insights with greater clinical relevance. Moreover, new LLM-driven methods for preprocessing and standardization may enhance interoperability with traditional electronic health records, expanding multimodality. Even without gold-standard annotations, the results achieved by this early effort merit further evaluation in public health settings with unmet needs, particularly in systems with limited capacity for other types of data collection. Despite limitations, this work highlights the potential of unscripted audio data to enable automation of tasks involving public health challenges beyond COVID-19.

## Methods

**Fig. 2:**
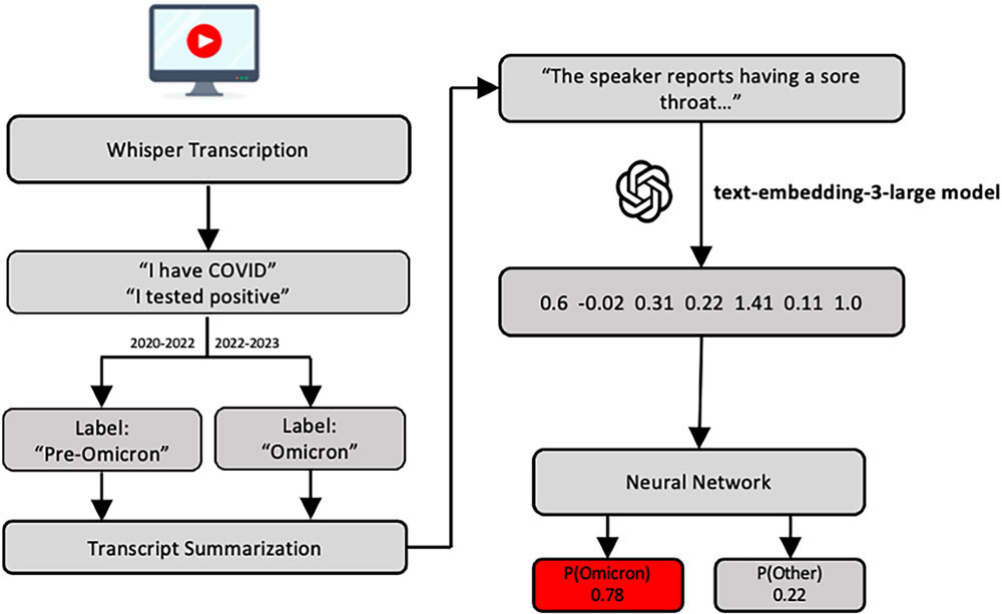
Overview of the AI pipeline used to preprocess and model unstructured audio data. The pipeline included the following steps: (1) The Whisper-Large model was used to transcribe the recorded first-person accounts of COVID-19 infections, (2) the o1 large language model was used to generate a filtered summary of the transcript, removing terminology that could have compromised the simulation of an early-stage outbreak, (3) the summaries were embedded using the text-embedding-3-large model, (4) a neural network was trained for variant classification.

This study was approved by the Institutional Review Board of the National Institutes of Health. Figure 2 illustrates the data preprocessing, and modeling components of the AI pipeline. All foundation models were accessed through the OpenAI API^29^.

### Dataset curation

To facilitate variant classification, YouTube videos about COVID-19 experiences were first manually verified to ensure that the speaker explicitly confirmed a current or previously positive diagnostic test and discussed their experiences/symptoms related to the illness. Videos that contained brief mentions of a positive test but no other health-related information were excluded from the study. The videos were then categorized as either “Omicron” (the new, emerging variant for the purposes of the simulation) or “Pre-Omicron” based on the date of the illness that was reported by the speaker or indicated by video metadata. This binary labeling method was selected due to the relative similarity between COVID-19 variants prior to Omicron. The Omicron variant represented a shift in symptom presentation and transmissibility. Omicron was designated a “variant of concern” on November 26th, 2021 by the World Health Organization, and was estimated to be the dominant variant in the U.S. by late December 2021^30,31^. Soon after, Omicron was identified as the dominant variant globally, accounting for over 98% of sequences shared on GISAID after February 2022^32,33^. Videos labeled “Omicron” were recorded on or after December 1st, 2021 and include both the original Omicron variant and subvariants. Videos recorded on or prior to November 30th, 2021, were labeled as “Pre-Omicron” (Table 4).

### Automatic speech recognition

Automatic speech recognition was performed using the Whisper-large model (OpenAI), which generated a transcript for each video^29,34^. Whisper had a low error rate of 11% on benchmark tasks derived from the Mozilla Common Voice dataset, potentially indicating reliability in diverse healthcare settings^35,36^. In the case of speakers who described a single experience with COVID-19 throughout the course of multiple videos, the transcripts were concatenated in chronological order to ensure that temporal context was preserved in the unified summary.

### Transcript summarization

To reduce dimensionality and noise, an LLM was instructed to summarize the COVID-19 case described by the speaker (Table 5). Summaries were generated in paragraph form to preserve descriptive language that may contain more nuanced biomarkers of factors like disease severity (e.g., “my very sore throat makes it hard to swallow water” instead of “sore throat”). Neither the o1 model nor the prompts were fine-tuned for this task, mirroring circumstances where the necessary compute and expertise may be unavailable to improve existing generative AI tools through advanced methods (i.e., beyond prompt engineering).

**Table 5:** Prompt used for LLM-driven summarization of audio transcripts about COVID-19

Analyze the following transcript and generate a detailed 1-paragraph summary of the speaker’s COVID-19 case that would be relevant to a clinician performing a health assessment. Guidelines: **Summarize** statements describing experiences related to physical and psychological symptomatology (e.g., ‘I feel short of breath’, ‘I have a fever’, ‘I couldn’t taste anything, ‘my throat is sore’). **Do not include** any information about names of people, dates (day, month, or year), COVID-19 variants, COVID-19 testing methods (e.g., LFDs, Rapid Tests, PCR tests), COVID-19 medications (e.g., Paxlovid), COVID-19 vaccination status, COVID-19 booster vaccination status, or COVID-19 reinfection history. **Do not include** the following words and phrases anywhere in the summary: “2020”, “2021”, “2022”, “2023”, “2024”, “omicron”, “delta”,“pcr test”, “rapid test”, “lfd test”, “antigen test”, “paxlovid”, “monoclonal antibodies”, “remdesivir”,“vaccine”, “vaccinated”, “vaccination status”, “unvaccinated”, “booster”, “boosted”,“first dose”, “second dose”, “third dose”, “reinfection”, “reinfected”, “second time”, “multiple infections” If no clinically relevant symptoms are mentioned in the transcript, return an empty string (“”).	

As shown in Table 5, the model was instructed to exclude variables that more frequently co-occurred with a specific predominant variant but were unrelated to health status, including dates, variant names, reinfection status, vaccination status, testing methods, and medications. For example, the peak period for the use of lateral flow devices (LFDs) occurred in early 2022, when Omicron was the prevalent variant^37^. As such, there was a higher probability of the term “rapid test” being mentioned in a YouTube video from the Omicron cohort (after Nov. 30th, 2021). However, the increased use of LFDs was due to technological innovation and expanded testing programs, not a change in COVID-19. Reinfections were also more common during the Omicron wave, partially due to decreased immunity over time and increased exposure following the discontinuation of prevention measures—factors that are extrinsic to the disease^38^. This preprocessing step was taken to ensure an accurate simulation of real-time AI usage in a newly developing public health crisis. In this context, the clinical reliability of unstructured audio data is best evaluated based on the ability to capture nuanced disease phenotypes. Due to the high variability of information density in YouTube videos, the model was also given the option to decide that the transcript contained insufficient health information, reducing the risk of noise in training data.

The o1 LLM, a newly released “chain-of-thought” model, was used to summarize the transcripts, achieving perfect accuracy when removing terminology that could co-occur with variant status (Table 5)^32^. This was in contrast to the performance of GPT-4o, which applies only a singular reasoning iteration before responding to instructions. The set of summaries generated by GPT-4o contained 16 instances of the words or phrases from Table 5, including 13 references to past infections. This information may be indicative of a variant that occurs later in time (i.e., Omicron) and could compromise the simulation of an emerging health crisis in which only symptomatology data is readily available.

### Data binarization

Prompts were also formulated to enable further reduction of the audio transcript summaries. The o1 model was instructed to extract binary information related to the presence or absence of 17 common COVID-19 symptoms (Table 6), mirroring the data used for “symptom checking” methods that were previously shown to obtain similar performance as audio AI methods^23^. Here, the LLM returned a binary list for each summary, representing the symtompataology of the COVID-19 infection. Such data is not unique to audio and could be derived from conventional eletronic health records or simple surveys.

**Table 6:** Prompt for LLM-driven binarization of COVID-19 symptoms referenced in audio transcripts

The task is to analyze a health summary provided as input and return a Python dictionary with the following symptoms as keys: 1. Runny nose 2. Fever 3. Loss of appetite 4. Loss of smell 5. Sore throat 6. Nausea 7. Headache 8. Diarrhea 9. Non-productive cough 10. Productive cough 11. Muscle aches 12. Fatigue 13. Shortness of breath 14. Joint pain 15. Chest pain 16. Loss of taste 17. Abdominal pain **For each symptom:** Set the value to ‘1‘ if the symptom is explicitly mentioned as being present at any point in the summary, even if the summary later mentions that the speaker is no longer experiencing this symptom (e.g., “The speaker described a sore throat” indicates a sore throat). Set the value to ‘1‘ if there are indirect references to the symptom (including any synonyms) and it is implied as being present (e.g., “The speaker described a scratchy throat” or “The speaker described a hoarse voice” indicates a sore throat). Set the value to ‘0‘ if the symptom is explicitly negated or stated as absent (e.g., “The speaker denied having a sore throat” or “The speaker was worried about a sore throat but did not have one”). Set the value to ‘0‘ if neither direct nor indirect references to the symptom are present in the summary. Be comprehensive in interpreting both direct and indirect references to symptoms, as well as absences of symptoms. Return only the dictionary in Python format.	

### Variant classification

#### Classification of embedded summaries

To facilitate variant classification, summaries were encoded into representation vectors by the text-embedding-3-large model (OpenAI), which was chosen because of a knowledge cutoff set in September 2021 (prior to the Omicron variant)^29^. This protected the efficacy of the simulation in which audio AI models were used to identify biomarkers of an emerging disease prior to the establishment of a robust knowledge base. Neural networks were then trained to predict the variant of COVID-19 that infected the speaker. To ensure compatibility with edge applications, the neural network model used in this study had only 787,202 trainable parameters – smaller than most AI algorithms for natural language processing or voice/speech tasks. As such, CPU were sufficient for training and evaluating these models. No high-performance computing resources were necessary for this study beyond those used by the providers of LLM APIs. The cross-entropy loss function was applied to assess model error after each minibatch of data (batch size of 8). Subsequent weight adjustments were performed using the Adam optimization algorithm with a learning rate of 1e-3. The extent of model training was determined through an early stopping protocol based on validation loss and a patience parameter of three. Training was concluded if there was no decrease in the validation loss for three consecutive epochs. Nested *k*-fold cross validation was used to evaluate model performance and generalizability.

#### Symptom assessment model

**Fig. 3:**
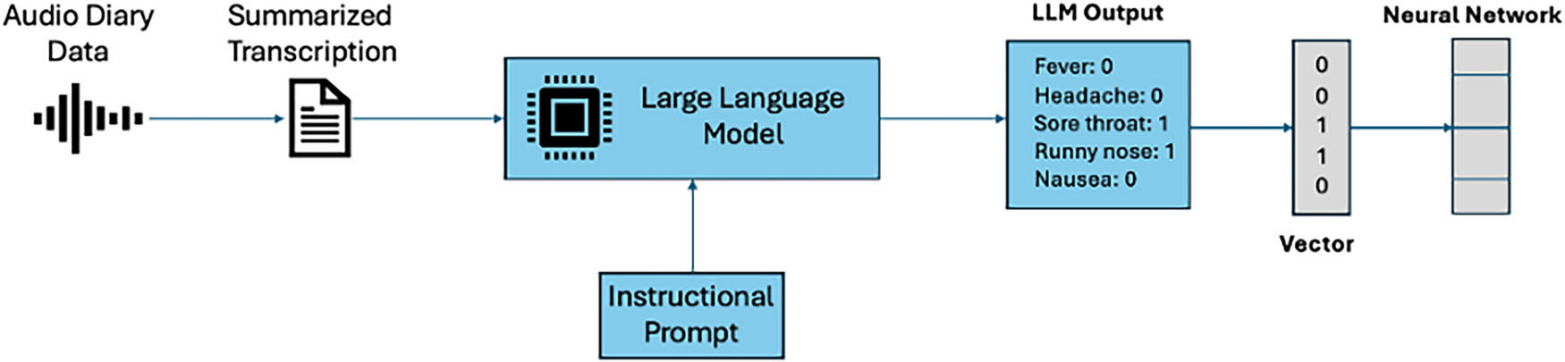
Overview of the pipeline used to binarize unstructured audio data. The pipeline included the following steps: (1) summarized transcripts were input into the o1 LLM, which converted the data into tabular binary format (presence/absence of symptoms), (2) neural networks were trained to perform variant classification on the binarized symptom data.

For comparision purposes, a second neural network model was trained on vectors of binarized symptoms (Table 6) designed to replicate conventional data for “symptom checking” (Figure 3). As above, the cross-entropy loss function was used to train the model on minibatches of data.

## Data Availability

Data supporting the findings of this study can be provided upon reasonable request to the corresponding author.

## Code availability

Code used in this study can be made available upon reasonable request to the corresponding author.
